# Circulating Tumor DNA Detection for Recurrence Monitoring of Stage I Non‐Small Cell Lung Cancer Treated With Microwave Ablation

**DOI:** 10.1111/1759-7714.15534

**Published:** 2025-01-18

**Authors:** Lin Cheng, Sheng Xu, Yu‐feng Wang, Sheng‐wei Li, Bin Li, Xiao‐Guang Li

**Affiliations:** ^1^ Department of Minimally Invasive Tumor Therapies Center Beijing Hospital, National Center of Gerontology, Institute of Geriatric Medicine, Chinese Academy of Medical Sciences Beijing China; ^2^ Medical School University of Chinese Academy of Sciences Beijing China

**Keywords:** ctDNA, efficacy, microwave ablation, NSCLC

## Abstract

**Purpose:**

As microwave ablation continues to be used in patients with inoperable stage I non‐small cell lung cancer (NSCLC), it is particularly important to monitor efficacy. Whether plasma ctDNA detection can predict its efficacy should be illustrated.

**Methods:**

We recruited 43 patients with inoperative stage I NSCLC, all of whom underwent biopsy‐synchronous microwave ablation (MWA). Peripheral blood samples were collected at baseline (*n* = 43), within 1 h post‐MWA (*n* = 28), and at the landmark time point (*n* = 26) for MRD detection. Clinical outcomes were analyzed using Kaplan–Meier survival analysis.

**Results:**

Patients with undetectable ctDNA at baseline (*p* = 0.042) and within 1 h after MWA (*p* = 0.023) had better clinical outcomes. In particular, patients with undetectable ctDNA at the 1‐h post‐MWA time point did not experience recurrence. Detection of ctDNA at the landmark time point is considered an independent risk factor for prognosis and is strongly correlated with clinical outcomes (*p* = 0.001), the median time to recurrence indicated by ctDNA was 4.9 months earlier compared to imaging. The clinical outcomes of patients with ctDNA clearance were similar to those with no ctDNA (*p* = 0.570). Risk stratification indicated that patients with persistent ctDNA had worse clinical outcomes compared to those who never had detectable ctDNA (*p* = 0.004).

**Conclusion:**

Our findings suggest that ctDNA monitoring can assist in predicting clinical outcomes in stage I NSCLC treated with microwave ablation. Patients with undetectable ctDNA within 1 h after MWA are determined to be clinically cured. Risk stratification based on ctDNA test results helps to differentiate high‐risk patients.

## Graphical abstract

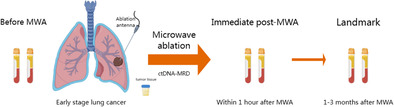

## 1 Introduction

Lung cancer is still the leading cause of cancer death worldwide, and its incidence is increasing year by year. According to the statistics of the National Cancer Institute of the United States in 2022, the 5‐year relative survival rate of lung cancer is 22.9% [1]. Surgery is the best curative method, but for patients with decreased physiological function or severe diseases who are difficult to tolerate or subjectively refuse high‐risk surgery, image‐guided ablation therapy represented by microwave ablation (MWA), as a tumor local treatment technology with good effect, precision and controllability, safety and repeatability, has become an alternative therapy for these patients. For patients with tumor size ≤ 3 cm, the efficacy and safety of ablation therapy have been confirmed by many studies [2], and it has been consistently recommended by international authoritative institutions such as the National Comprehensive Cancer Network (NCCN) and the Cardiovascular and Interventional Radiological Society of Europe (CIRSE) as an alternative to surgery or stereotactic radiotherapy. However, compared with surgery, MWA has a higher rate of local recurrence, so routine post‐MWA followup is important to detect early recurrence.

Currently, the efficacy evaluation of MWA routinely involves computed tomography (CT) follow‐up every 3 months postoperatively. But, for patients with low recurrence risk, receiving unnecessary imaging exams increases the risk of radiation damage and economic burden, and for patients with high recurrence risk, the sensitivity of imaging in detecting residual tumor recurrence is insufficient. Therefore, it is necessary to explore new methods for evaluating the efficacy of post‐ablation.

In recent years, the application of liquid biopsy represented by circulating tumor DNA (ctDNA) has been gradually increasing. As a promising biomarker for the detection of molecular residual disease (MRD) in cancer, ctDNA has been studied for early diagnosis, prognostic stratification, disease monitoring and treatment response assessment [3, 4, 5, 6, 7]. Several studies have shown that the persistence of ctDNA is closely associated with recurrence, metastasis, and prognosis after surgery of lung cancer [8, 9, 10].

However, the treating mechanism of MWA is different from that of surgery in that it mainly inactivates tumor tissue in situ by high temperature, resulting in coagulation and necrosis of the tumor tissue. Theoretically, the dynamics of ctDNA after MWA should be different from surgery, and how it changes is unclear. To our knowledge, there are no studies that have used ctDNA detection as an adjuvant to predict the efficacy of patients with stage I NSCLC treated with MWA.

## 2 Materials and Methods

### 2.1 Study Design

The study protocol and informed consent form were approved by the Ethics Review Committee of Beijing Hospital [Ethics Approval No.: 2022BJYYEC‐420‐02]. The study was conducted in accordance with the Declaration of Helsinki. Written informed consent was obtained from all patients. This observational study began recruiting stage I NSCLC patients undergoing microwave ablation at Beijing Hospital in August 2022. Inclusion Criteria: (i) Age > 18 years; (ii) patients with pathologically confirmed non‐small cell lung cancer and stage I according to the International Association for the Study of Lung Cancer 8th edition TNM staging; (iii) patients with an Eastern Cooperative Oncology Group Performance Status (ECOG PS) score of 0–2; (iv) patients cannot tolerate or refuse surgery and radiotherapy due to advanced age, severe comorbidities (e.g., coronary artery disease, interstitial fibrosis), and other reasons; (v) expected survival of more than 3 months. Exclusion criteria: (i) Patients who suffering from other malignant tumors or severe multi‐organ dysfunction; (ii) expected survival less than 3 months; (iii) unable to accept or provide designated means of efficacy assessment such as PET‐CT or contrast‐enhanced CT.

Progression‐free survival (PFS) was defined as the time from the day of MWA to the first imaging‐confirmed recurrence or death. Typically, nodular or central enhancement > 10 mm and/or > 15 HU on contrast‐enhanced CT was considered a local recurrence.

### 2.2 Sample Collection

**FIGURE 1:**
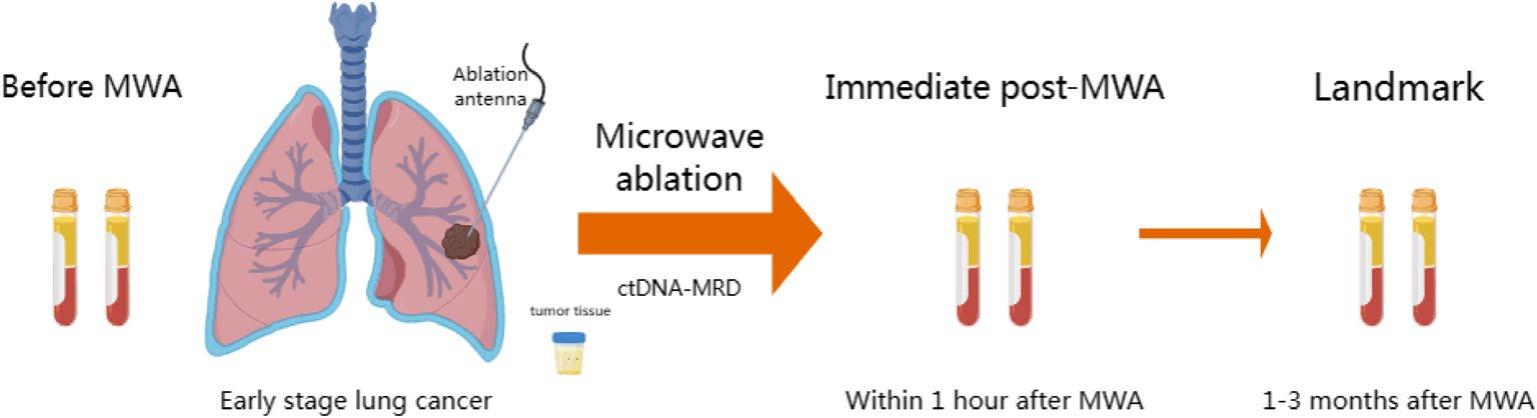
A flowchart depicting the blood sampling time points.

We obtained biopsy tumor tissue prior to MWA. Peripheral blood samples (20 mL) were collected 24 h before MWA, within 1 h post‐MWA, and 1–3 months post‐MWA, and stored in Streck tubes (Figure 1). All blood samples were analyzed within 3 days of collection. The landmark time point was defined as 1–3 months post‐MWA.

### 2.3 Sample Processing and ctDNA Detected

Puncture tumor specimens were prepared as formalin‐fixed, paraffin‐embedded (FFPE) for DNA extraction using the QIAamp DNA Mini Kit (Qiagen, Hilden, Germany). DNA quantity and integrity were measured with a Qubit 2.0 Fluorometer (Life Technologies) and a 2100 Bioanalyzer (Agilent). Peripheral blood samples, collected in Streck tubes, were centrifuged at 1600 g for 10 min, then 16 000 g for 10 min. The plasma cfDNA and peripheral blood leukocytes (PBLs) were separated and frozen at −80°C. CfDNA was extracted using the QIAamp Circulating Nucleic Acid Kit (Qiagen, Hilden, Germany), and germline genomic DNA from PBLs using the QIAamp DNA Blood Mini Kit (Qiagen, Hilden, Germany). DNA quantity and integrity were again measured with the Qubit 2.0 Fluorometer (Life Technologies, CA, USA) and the 2100 Bioanalyzer (Agilent, CA, USA).

Tumor DNA and germline genomic DNA were fragmented to 200–250 bp using the Bioruptor UCD‐200 (Diagenode, NJ, USA). For library construction, 20–80 ng of cfDNA was used, and unique identifiers (UIDs) were tagged on each double‐stranded DNA to distinguish authentic somatic mutations from artifacts. Indexed NGS libraries were prepared using the KAPA Library Preparation Kit (Kapa Biosystems, Wilmington, MA, USA).

Target DNA enrichment for tumor and paired germline was done using a custom panel covering ~1.5 Mb coding sequences of 1021 cancer‐related genes (Geneplus, Beijing, China). Plasma and paired germline DNA were enriched using a biotinylated probe targeting 338 cancer‐related genes over 550 kb (Geneplus, Beijing, China). Sequencing was performed on a DNBSEQ‐T7RS sequencer (MGI Tech, Shenzhen, China) with 100‐bp paired‐end reads.

FASTP [11] was used for quality control of FASTQ files, removing adapters, low‐quality reads, and N bases. Reads were mapped to the human genome (hg19) using Burrows–Wheeler Aligner [12]. Duplicates were removed with Picard's MarkDuplicates tool (version 4.0; Broad Institute). GATK (v3.6‐0‐g89b7209; Broad Institute) called somatic single‐nucleotide variants (SNVs) and indels, CONTRA (2.0.8) identified copy number variations (CNVs) [13], and NCSV (v0.2.3; GeneplusBeijing, inhouse) detected structural variants (SVs). Variants were manually confirmed using the Genomics Viewer. Germline variants were screened in dbSNP and population databases like ExAc (v0.3.1) and 1000 Genomes.

UID and REALSEQ (v3.1.0; Geneplus‐Beijing, inhouse) removed duplicate reads and sequencing errors, with SNVs and indels called using REALDCALLER (v1.8.1; Geneplus‐Beijing, inhouse) and GATK. TNSCOPE (Sentieon Inc., SanJose, CA, USA) improved detection of long indels. cfDNA variants were filtered out as references [9]. Plasma cfDNA variants were classified as “tissue‐derived” if present in matched tumor tissue, or “ctDNA private” if not. Variants were considered true somatic mutations under specific conditions: tissue‐derived driver mutations with ≥ 2 reads, nondriver mutations with ≥ 4 reads, ctDNA private driver mutations with ≥ 4 reads, and nondriver mutations with ≥ 8 reads. A plasma sample with at least one detected variant was defined as ctDNA‐positive.

### 2.4 MW Ablation Procedure

**FIGURE 2:**
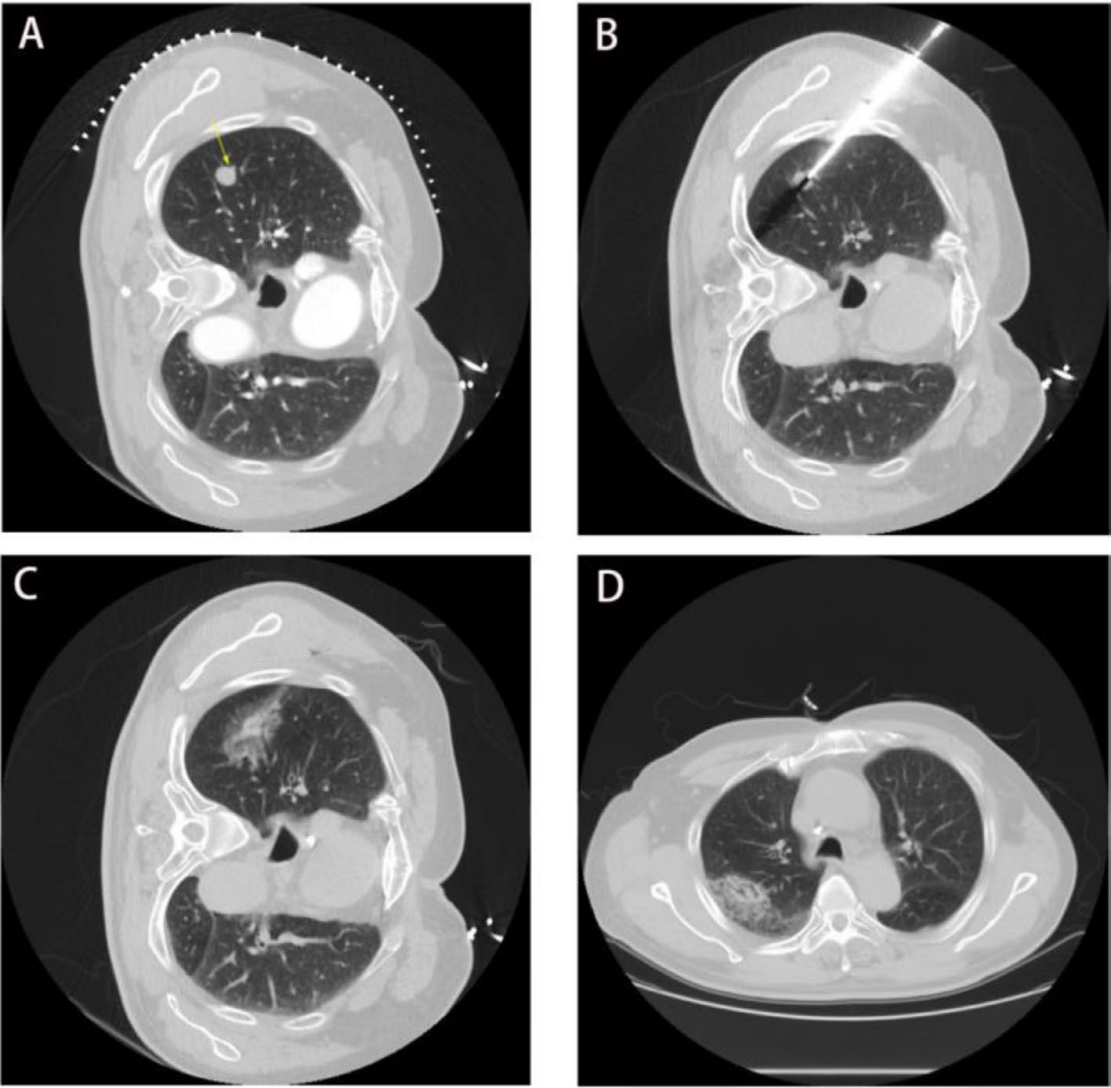
Typical case of microwave ablation procedure. (A) CT scan localization. (B) Ablation antenna in lesion. (C) Image at the end of microwave ablation. (D) Next‐day after microwave ablation.

All patients underwent chest CT and/or contrast‐enhanced CT (CT590; GE Healthcare, Pittsburgh, Pennsylvania) to assess the location, number, and size of tumors. The MWA indication and procedure follow the guidelines of the Society of Interventional Radiology [14, 15]. The MW ablation antennas (Vision Medical or ECO Medical Instrument, Nanjing, China) were 15–18 cm in effective length and 15–17 gauge of outer diameter according to the tumor location and distance to the pleura, with a 15‐mm active tip. Ablation was performed under CT by two radiologists with more than 5 years of experience of tumor ablation, using the MTC‐3C MW ablation system (Vison Medical, Nanjing, China) or the ECO‐100A1 MW ablation system (ECO, Nanjing, China) (microwave emission frequency: 2450 ± 50 MHz, The adjustable continuous wave output power: 20–80 W). To ensure complete ablation of the lesion, we performed multi‐site ablation, using two microwave antennas simultaneously when necessary. Ablation was stopped when a 5–10 mm ground‐glass opacity appeared beyond the lesion's boundary. A follow‐up CT scan was done the next day to check for adverse reactions such as pneumothorax or bleeding (Figure 2).

### 2.5 Statistical Analysis

Categorical variables were described as frequencies and percentages and were compared by χ^2^ test. Continuous variables are described as mean ± SD and were compared by independent samples Student's *t* test. Kaplan–Meier analyses were used to describe survival outcomes, and risk ratios were compared using the log rank test for comparison of survival outcomes. *p* ≤ 0.05 was considered a statistically significant difference. Statistical analysis was performed using SPSS 26.0 (IBM Corp., USA).

## 3 Results

### 3.1 Patient Characteristics

As of January 2024, a total of 43 patients with stage I NSCLC were included in the study. All patients successfully underwent percutaneous biopsy combined with synchronous MWA. Histopathological examination confirmed the presence of adenocarcinoma in 33 patients (76.7%), squamous cell carcinoma in 8 patients (18.6%), and other pathological types in 2 patients (4.7%). The average age of the patients was 72.93 ± 8.22 years, with 93.0% (*n* = 40) being over 60 years old. Additionally, 74.4% of the patients had comorbidities, and 44.2% (*n* = 19) had a history of smoking. Among them, 72.1% (*n* = 31) were in stage IA. After MWA, 14% (*n* = 6) of the patients developed pneumothorax, and 1 patient experienced intrapulmonary hemorrhage. Other patient characteristics are detailed in Table 1. By the end of the follow‐up period, 11 patients (25.6%) had experienced a recurrence (including 6 local recurrences and 5 distant metastases). The median follow‐up duration was 13.9 months (range: 3.8–27.5 months).

**TABLE 1:** Baseline characteristics of enrolled patients.

Variables	Patients (*n* = 43)
Age (y)	72.93 ± 8.22
> 60	40 (93.0%)
< 60	3 (7.0%)
Gender	
Male	25 (58.1%)
Female	18 (41.9%)
Comorbidity	
Yes	32 (74.4%)
No	11 (25.6%)
ECOG	
0	25 (58.1%)
1	15 (34.9%)
2	3 (7.0%)
Smoking history	
Yes	19 (44.2%)
No	24 (55.8%)
Tumor size(cm)	2.35 ± 0.84
Tumor stage	
IA	31 (72.1%)
IB	12 (27.9%)
Location of the tumor	
Left lung	19 (44.2%)
Right lung	24 (55.8%)
Tumor pathology	
Adenocarcinoma	33 (76.7%)
Squamous cell carcinoma	8 (18.6%)
Others	2 (4.7%)
Histological grading	
G1	4 (9.3%)
G2	22 (51.2%)
G3	17 (39.5%)
Number of MWA antennas	
1	34 (79.1%)
2	9 (20.9%)
Adverse event	
Pneumothorax	6 (14.0%)
Pulmonary hemorrhage	1 (2.3%)
Laboratory indicators	
CA199 (U/ml)	13.94 ± 20.43
CA125 (U/ml)	15.05 ± 8.44
CEA (ng/ml)	9.60 ± 17.54
SCCA (ng/ml)	1.37 ± 2.14

This study successfully analyzed 43 pre‐MWA blood samples, 41 tumor tissue samples, 28 blood samples collected within 1 h after MWA, and 26 landmark blood samples. In tissue mutations, a total of 585 variants were identified in tumor tissue, with an average mutation frequency of 203.96% per patient. Somatic mutations included 431 point mutations/small fragment insertions‐deletions, 93 amplifications, 49 deletions, and 8 fusions. Additionally, there were 4 germline mutations. Among these somatic mutations, there were 29 Class I mutations (4.96%), 105 Class II mutations (17.95%), and 447 Class III mutations (76.41%). Among the 41 tumor tissue samples, EGFR mutations were observed in 24 patients, TP53 mutations in 24 patients, LRP1B mutations in 10 patients, and KRAS mutations in 9 patients. These four mutations were the most frequently observed.

### 3.2 The Predictive Value of Detecting ctDNA Before MWA

**FIGURE 3:**
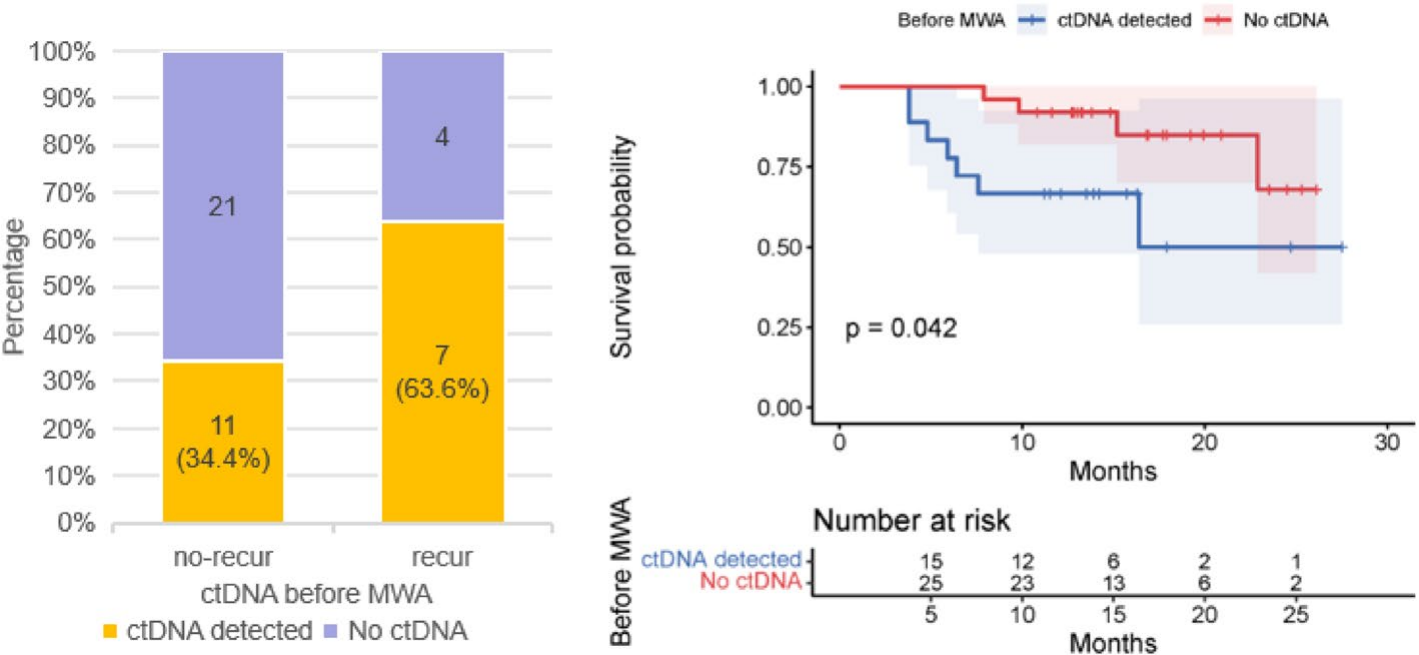
The distribution of ctDNA positivity and Kaplan–Meier survival analysis of ctDNA‐detected patients and no‐ctDNA patients.

In the pre‐MWA cohort, ctDNA was detected in the plasma of 18 patients (41.9%). The detection rate was 35.5% in stage IA patients (*n* = 31) and 58.3% in stage IB patients (*n* = 12), as shown in Figure S1A. The detection rate of ctDNA was significantly higher in squamous cell carcinoma (LUSC) at 75%, compared to 30.3% in adenocarcinoma (LUAD) (Figure S1B). The detection rate of ctDNA increased with higher histological grades (G1: 0%; G2: 27.3%; G3: 70.6%, Figure S1C). There was no significant difference in ctDNA positivity rates among the four common mutations (EGFR, TP53, LRP1B, KRAS, Figure S2). However, ctDNA was more likely to be positive in patients with TP53 mutations (*p* = 0.027, Table S1). Other baseline characteristics showed no significant differences (Table S1). At the pre‐MWA time point, 63.6% of patients who had recurred were ctDNA positive, and only 34.4% of patients who had not recurred were ctDNA positive (Figure 3). Kaplan–Meier survival analysis was performed to determine clinical outcomes. We found that the PFS of ctDNA‐detected patients was superior to that of no‐ctDNA patients (*p* = 0.042, Figure 3). The 1‐year PFS rates for ctDNA‐detected and no‐ctDNA patients were 0.67 and 0.92, respectively.

### 3.3 The Predictive Value of Detecting ctDNA After MWA

To observe how ctDNA changes in patients after MWA treatment, we collected peripheral blood samples at two time points: within 1 h post‐MWA and at 1–3 months post‐MWA (landmark). Within 1 h post‐MWA, we collected 28 peripheral blood samples, with a ctDNA‐positivity rate of 60.7%. At the landmark, we collected 26 peripheral blood samples, with a ctDNA‐positivity rate of 34.6%. The trend in ctDNA detection rates at the three time points is shown in Figure S3. Additionally, we found that if ctDNA was detectable before MWA, it was also detectable within 1 h post‐MWA.

**FIGURE 4:**
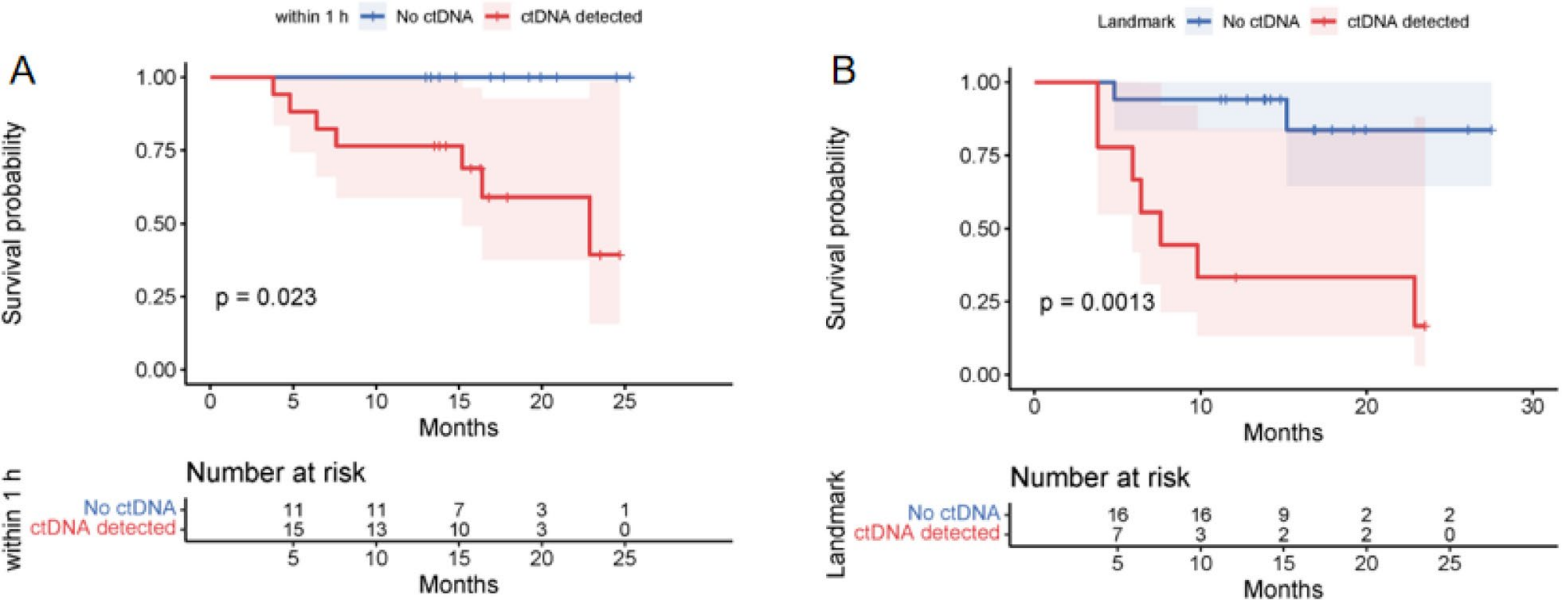
The Kaplan–Meier survival analysis of ctDNA‐detected patients and no‐ctDNA patients within 1 h after MWA (A) and at landmark (B).

To explore the reasons for the increased positivity rate of ctDNA within 1 h post‐MWA, we compared tumor characteristics, MWA power, and duration between patients with detected ctDNA and those without. We found that larger tumor size, stage IB, higher histological grade, and higher MWA power were associated with a greater likelihood of ctDNA shedding into the bloodstream (Table S2). At this time point, all of the patients (100%) who had recurred were ctDNA‐positive, and 47.6% of the patients who had not recurred were ctDNA‐positive. To determine whether ctDNA detection within 1 h post‐MWA predicts clinical outcomes, Kaplan–Meier survival analysis was performed. We found that no‐ctDNA patients have significantly better PFS compared with ctDNA‐detected patients (*p* = 0.023, Figure 4A), and the 1‐year PFS rates were 1 versus 0.76, respectively.

We assume that patients with detectable ctDNA at landmark are considered at high risk of recurrence. Therefore, to clarify the baseline characteristics of these patients, we conducted comparative analysis. We also compared the working parameters of the ablation antenna between patients with detectable ctDNA and those without to rule out any potential influence. And we found that the detection of ctDNA at landmark is considered an independent prognostic risk factor, unaffected by baseline characteristics, working parameters of the ablation antenna, or tissue mutations (Table S3). At the landmark time point, 77.8% of patients who had recurred were ctDNA positive, and only 11.8% of patients who had not recurred were ctDNA positive. We conducted Kaplan–Meier survival analysis and found that patients with no ctDNA had significantly better PFS compared with those with ctDNA detected (*p* = 0.0013, Figure 4B), with a 1‐year PFS rate of 0.94 versus 0.33, respectively. Among patients who were ctDNA positive and experienced recurrence at the landmark time point, the median time to recurrence indicated by ctDNA was 4.9 months (range 1–13.5 months) earlier compared to imaging.

### 3.4 ctDNA Clearance Defines Patient With Lower Risk or Recurrence

**FIGURE 5:**
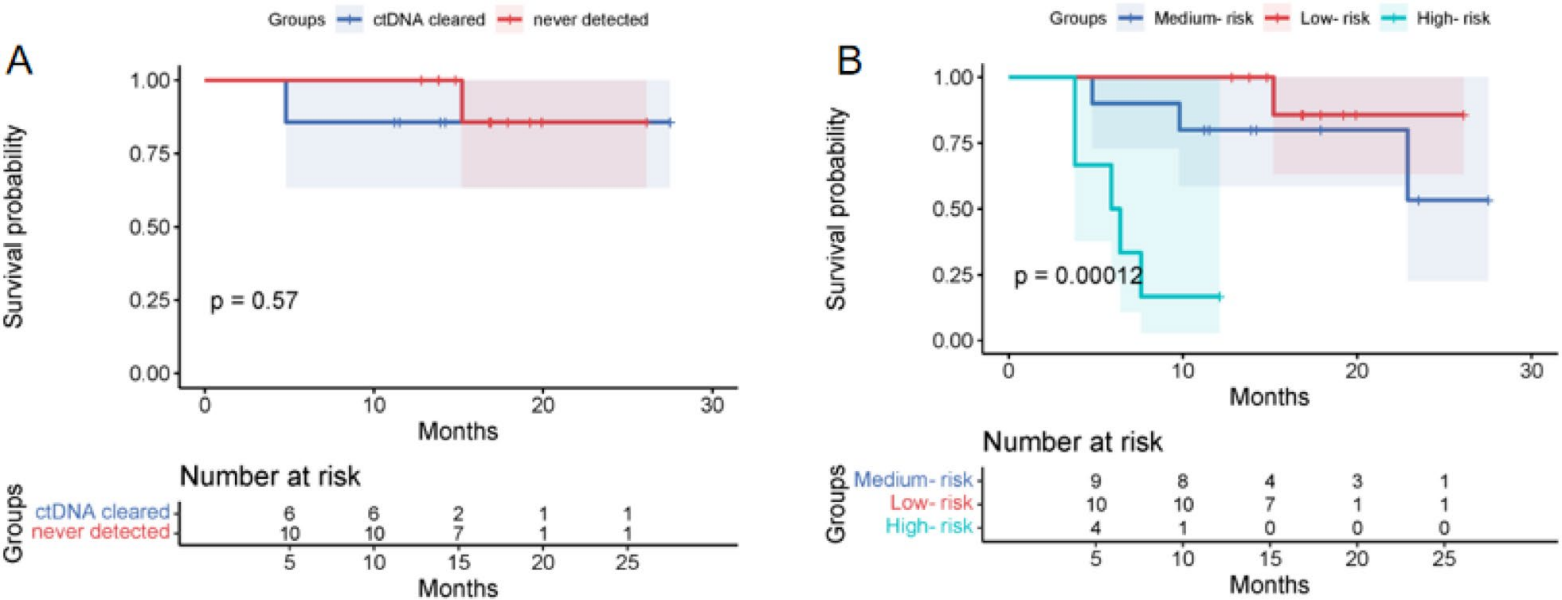
(A) The Kaplan–Meier survival analysis of ctDNA‐clearance patients and no‐ctDNA patients. (B) The Kaplan–Meier survival analysis of low‐risk, medium‐risk, and high‐risk group.

We defined “clearance” of ctDNA as a transition from ctDNA‐positive at baseline to ctDNA‐negative at landmark after MWA treatment. Among the 13 patients who were ctDNA‐positive at baseline, seven patients (53.8%) converted to ctDNA‐negative and only 1 patient has experienced recurrence. There was no significant difference in PFS between patients with ctDNA clearance and those with no ctDNA (*p* = 0.570, Figure 5A). We categorized patients into three groups (Figure S4) based on ctDNA clearance: low‐risk group (*n* = 10)—ctDNA was not detected at both baseline and landmark; medium‐risk group (*n* = 10)—ctDNA was detected either at baseline or landmark; high‐risk group (*n* = 6)—ctDNA was detected at both baseline and landmark. We performed K–M survival analysis and found that, the medium‐risk group did not show inferior PFS compared to the low‐risk group (*p* = 0.341, Table S4), and the high‐risk group had significantly worse PFS (*p* < 0.001, Figure 5B). The PFS rates for the low‐risk, medium‐risk, and high‐risk group were 0.9 versus 0.7 versus 0.17, respectively.

To determine whether ctDNA clearance after MWA has prognostic value, we divided the patients who were tested for ctDNA into three groups: patients with no detectable ctDNA within 1 h post‐MWA and at the landmark time point (low‐risk group, *n* = 4); patients with detectable ctDNA within 1 h post‐MWA but not at the landmark time point (medium‐risk group, *n* = 6); patients with detectable ctDNA within 1 h post‐MWA and at the landmark time point (high‐risk group, *n* = 5). Due to the small number of people in each group, we only compared the recurrence rate, the PFS rate in the low risk group versus medium risk group vs. high risk group was 1 versus 0.67 versus 0.2, respectively (Figure S5).

### 3.5 The Predictive Value of Detecting ctDNA at all Three Time Point

**FIGURE 6:**
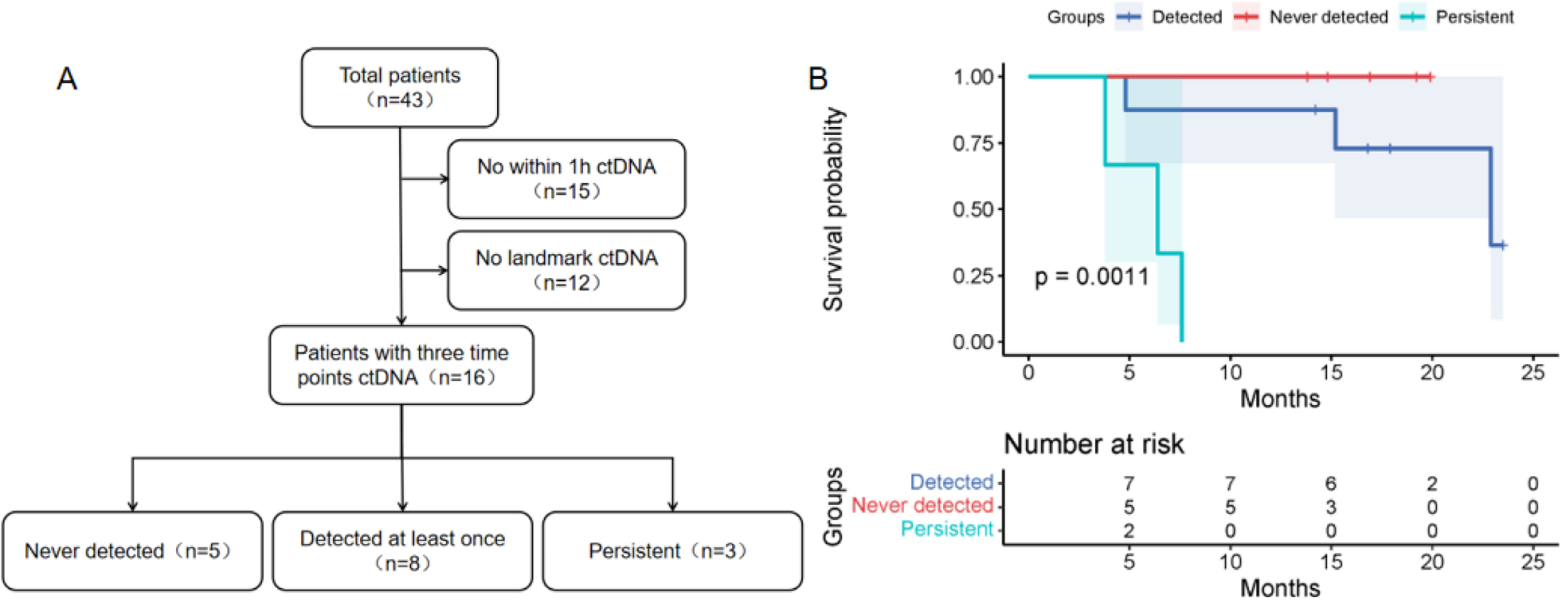
(A) Flowchart of patients analyzed for ctDNA detected at all three time points. (B) The Kaplan–Meier survival analysis of never detected ctDNA, detected ctDNA at least once, and ctDNA persistent groups.

In order to explore the value of longitudinal detection of ctDNA, three blood collection time points were set up in this study, but some patients were unable to complete the test within the specified time due to their own or objective reasons., We categorized patients who underwent ctDNA testing at all 3 time points (Figure 6A) into 3 groups: never detected ctDNA; detected ctDNA at least once; and ctDNA persisted. We performed K–M survival analysis and found that, compared with the never detected group, the detected at least once group did not show a significant inferior PFS (*p* = 0.289, Table S5), while the persistent group had significantly worse PFS (*p* = 0.004, Figure 6B). The PFS rates for the three groups were 1, 0.625, and 0, respectively (Figure S6).

## 4 Discussion

In our study, the majority of patients undergoing microwave ablation were over 60 years old (93%) or had comorbidities (74.4%), indicating microwave ablation as a treatment option for stage I NSCLC unsuitable for surgery. However, microwave ablation does not involve lymph node dissection and may have a higher recurrence rate compared to surgery, necessitating recurrence monitoring for these patients. Our study found that among 13 patients with ctDNA positive at baseline, 7 patients (53.8%) had their ctDNA clearance at the landmark time point, suggesting initial evidence of tumor clearance by microwave ablation. Moreover, patients with ctDNA clearance showed similar PFS to those with no ctDNA, consistent with previous research [16, 17]. We set a time point of 1 h post‐MWA in order to explore the effect of microwave ablation on plasma ctDNA and the prognostic value of ctDNA at that time point, we observed that if ctDNA was detectable before MWA, it was also detectable within 1 h post‐MWA (100%). This suggests that patients predisposed to shedding ctDNA into the bloodstream before treatment may have accelerated ctDNA release due to microwave ablation, potentially linked to larger tumor size and higher ablation power. Additionally, we found that patients who did not have detectable ctDNA within 1 h post‐MWA also did not show detectable ctDNA at the landmark time point, and no recurrence in any of these patients. This finding may suggest that patients with undetectable ctDNA within 1 h after MWA could be defined earlier as clinically cured patients.

Overall, the detection rate of ctDNA in stage I NSCLC was 41.9%, which is roughly comparable to the reported in the publications [10, 18]. The positivity rate of ctDNA in LUSC (75%) was higher than in LUAD (32.35%), consistent with the “shedding hypothesis” described by TRACERx study and Zhang et al. [19, 20]. Moreover, preoperative ctDNA likely originates predominantly from necrotic tumor cells in the primary tumor rather than from biologically active micrometastatic deposits. Based on this, postoperative ctDNA positivity correlates significantly with poorer recurrence‐free survival in LUSC patients [21], as it may better indicate residual tumor cells by that time. The detection rate of ctDNA within 1 h post‐MWA treatment was 60.7%. At the landmark time point, the detection rate of ctDNA was 34.6%. Our study reports, for the first time, plasma ctDNA changes in patients with inoperable stage I NSCLC treated with microwave ablation, providing data to support future studies in this area.

In this study, we included only stage I NSCLC patients. The undetectable ctDNA before MWA does not affect its use for monitoring recurrence after MWA. The 1‐year PFS rates for patients with ctDNA detected at baseline and within 1 h post‐MWA were both lower than those for patients with no ctDNA, and survival analysis also showed meaningful clinical outcomes. Interestingly, patients with undetectable ctDNA at the 1‐h post‐MWA time point did not experience recurrence. Detection of ctDNA at the landmark time point can be considered an independent prognostic factor, unaffected by tissue mutations and tumor characteristics. Moreover, the presence of ctDNA at the landmark time point is strongly associated with poorer clinical outcomes, suggesting that this practical endpoint could aid in comparing different treatment strategies and allow for more personalized, ctDNA‐guided therapy.

We divided patients into three risk groups based on ctDNA detection results. Compared with the low‐risk group, the medium‐risk group did not show worse PFS, but the high‐risk group had significantly poorer PFS. Patients with longitudinally undetectable ctDNA had a better PFS. This method of dividing risk subgroups facilitates a more precise stratification of patients and identifying high‐risk patients.

This study has some limitations. First, our study included a small number of cases (*n* = 43) and had a short follow‐up period. Second, we only set two blood sampling points post‐MWA, lacking longitudinal monitoring. Third, since we obtained biopsy samples simultaneously with ablation, the local sampling might not represent the entire tumor due to tumor heterogeneity, which could lead to inconsistencies [22]. Finally, ctDNA is considered to reflect systemic mutation characteristics. Many studies have reported that unique mutations detected in ctDNA are more numerous than those in tumor tissue [23, 24, 25], making it challenging to exclude false positives among these cfDNA mutations.

In conclusion, this observational study confirmed that ctDNA monitoring can assist in predicting clinical outcomes in stage I NSCLC treated with microwave ablation. Patients with undetectable ctDNA within 1 h after MWA are determined to be clinically cured. Risk stratification based on ctDNA test results helps to differentiate high‐risk patients.

## Author Contributions

**Lin Cheng:** data curation; formal analysis; methodology; project administration; software; writing – original draft. **Sheng Xu, Yu‐Feng Wang, and Sheng‐Wei Li:** implement microwave ablation; data curation. **Bin Li:** implement microwave ablation. **Xiao‐Guang Li:** funding acquisition; project administration; supervision; review and editing; corresponding author.

## Ethics Statement

The study was approved by the Beijing Hospital Ethics Committee (No. 2022‐BJYYEC‐420‐02).

## Consent

Written informed consent was obtained from all patients. Its publication is approved by all authors.

## Conflicts of Interest

The authors declare no conflicts of interest.

## Supporting information


Data S1.

## Data Availability

The data that supports the findings of this study are available in the Supporting Information of this article.
