# Supplementary material for: Circulating Tumor DNA Detection for Recurrence Monitoring of Stage I Non‐Small Cell Lung Cancer Treated With Microwave Ablation
Source: Thorac Cancer. 2025 Jan 18;16(2):e15534. doi: 10.1111/1759-7714.15534 (PMC11742128; doi:10.1111/1759-7714.15534)
Supplement: Supplementary file 1 — Data S1. [file TCA-16-e15534-s001.docx]

Circulating Tumor DNA Detection for Recurrence Monitoring of Stage I Non-small Cell Lung Cancer Treated with Microwave Ablation

Supplementary Information

**Supplementary Figure 1** Detection of circulating tumor (ctDNA) at baseline before MWA (A) according to clinical stage, (B) according to tumor pathology, and (C) according to histological grading.


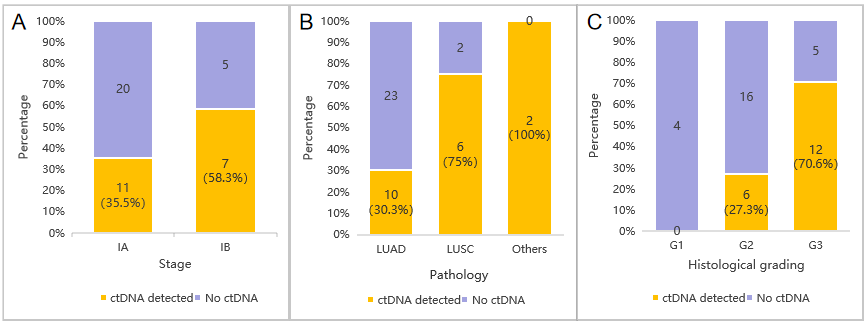


**Supplementary Figure 2** Detection of circulating tumor (ctDNA) at baseline before MWA by detected driver mutation.


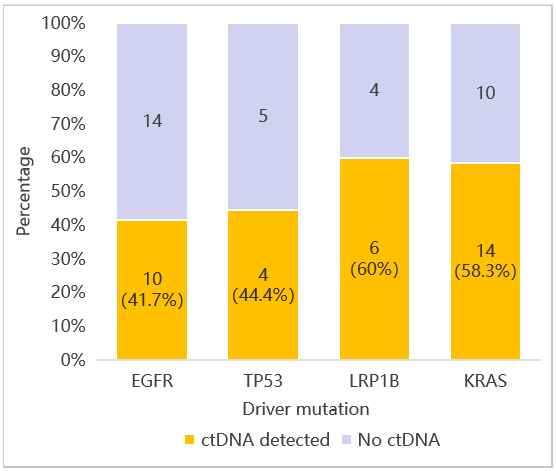


**Supplementary Table 1** Comparison of Clinical Baseline Characteristics Between ctDNA Detected and No ctDNA Patients Before Microwave Ablation.

| Variables | ctDNA detected(n=18) | No ctDNA(n=25) | *P* value |
| --- | --- | --- | --- |
| Age（y） | 73.83±7.06 | 72.28±9.04 | 0.547 |
| Gender |  |  | 0.112 |
| Male | 13 | 12 |  |
| Female | 5 | 13 |  |
| Smoking history |  |  | 0.203 |
| Yes | 10 | 9 |  |
| No | 8 | 16 |  |
| Comorbidity |  |  | 0.136 |
| Yes | 16 | 16 |  |
| No | 2 | 9 |  |
| Location of the tumor |  |  | 0.515 |
| left lung | 9 | 10 |  |
| right lung | 9 | 15 |  |
| Tumor size（cm） | 2.64±0.81 | 2.14±0.81 | 0.051 |
| Tumor stage |  |  | 0.173 |
| IA（≤3cm） | 11 | 20 |  |
| IB（＞3cm） | 7 | 5 |  |
| Tumor pathology |  |  | 0.018 |
| adenocarcinoma | 10 | 23 |  |
| Squamous cell carcinoma | 6 | 2 |  |
| Others | 2 | 0 |  |
| Histological grading |  |  | 0.002 |
| G1 | 0 | 4 |  |
| G2 | 6 | 16 |  |
| G3 | 12 | 5 |  |
| Ki67（%） | 38.61±23.69 | 10.17±10.00 | 0.000 |
| TMB（Muts/Mb） | 8.65±7.13 | 5.91±7.90 | 0.257 |
| Driver mutants |  |  |  |
| EGFR | 10 | 14 | 0.732 |
| TP53 | 14 | 10 | 0.027 |
| LRP1B | 6 | 4 | 0.416 |
| KRAS | 4 | 5 | 1 |
| Laboratory Indicators |  |  |  |
| CA199（U/ml） | 11.33±11.63 | 15.83±25.02 | 0.483 |
| CA125（U/ml） | 16.86±9.94 | 13.75±7.10 | 0.239 |
| CEA（ng/ml） | 15.58±25.47 | 5.28±5.70 | 0.057 |
| SCCA（ng/ml） | 2.04±3.21 | 0.88±0.38 | 0.081 |

**Supplementary Figure 3** The trend in ctDNA detection rates at three time points.


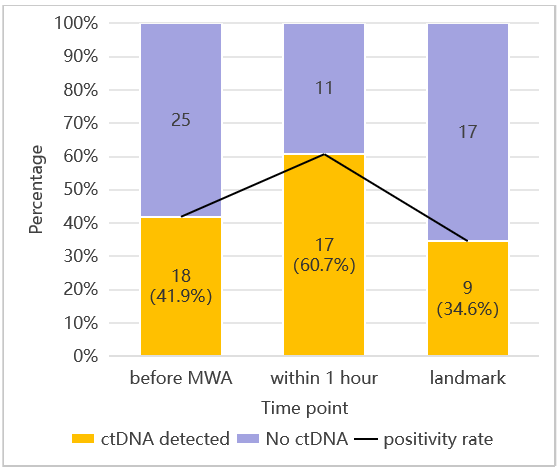


**Supplementary Table 2** Comparison of clinical baseline characteristics and ablation parameters between ctDNA detected and no ctDNA patients within 1-hour post-MWA.

| Variables | ctDNA detected(n=17) | No ctDNA(n=11) | *P* value |
| --- | --- | --- | --- |
| Age（y） | 74.06±8.23 | 75.64±7.58 | 0.614 |
| Gender |  |  | 0.488 |
| Male | 10 | 5 |  |
| Female | 7 | 6 |  |
| Smoking history |  |  | 0.867 |
| Yes | 8 | 4 |  |
| No | 9 | 7 |  |
| Location of the tumor |  |  | 0.142 |
| Left lung | 6 | 7 |  |
| Right lung | 11 | 4 |  |
| Tumor size（cm） | 2.86±0.75 | 1.68±0.53 | 0.000 |
| Tumor stage |  |  | 0.012 |
| IA（≤3cm） | 8 | 11 |  |
| IB（＞3cm） | 9 | 0 |  |
| Tumor pathology |  |  | 0.191 |
| Adenocarcinoma | 10 | 10 |  |
| Squamous cell carcinoma | 6 | 1 |  |
| Others | 1 | 0 |  |
| Histological grading |  |  | 0.019 |
| G1 | 0 | 3 |  |
| G2 | 8 | 6 |  |
| G3 | 9 | 2 |  |
| TMB（Muts/Mb） | 10.66±7.97 | 5.33±9.41 | 0.140 |
| Driver mutants |  |  |  |
| EGFR | 10 | 4 | 0.775 |
| TP53 | 14 | 4 | 0.122 |
| LRP1B | 6 | 0 | 0.123 |
| KRAS | 3 | 4 | 0.317 |
| Ablation power（W） | 36.76±5.57 | 29.09±4.37 | 0.001 |
| Ablation time（min） | 7.76±3.03 | 7.09±2.21 | 0.531 |
| Laboratory Indicators |  |  |  |
| CA199（U/ml） | 17.02±26.78 | 7.73±6.95 | 0.273 |
| CA125（U/ml） | 17.24±11.08 | 11.05±5.62 | 0.100 |
| CEA（ng/ml） | 13.05±23.21 | 3.89±3.50 | 0.208 |
| SCCA（ng/ml） | 1.25±1.04 | 0.85±0.36 | 0.231 |

**Supplementary Table 3** Comparison of clinical baseline characteristics and ablation parameters between ctDNA detected and no ctDNA patients at landmark.

| Variables | ctDNA detected(n=9) | No ctDNA(n=17) | *P* value |
| --- | --- | --- | --- |
| Age（y） | 74.44±7.92 | 71.06±7.08 | 0.276 |
| Gender |  |  | 0.257 |
| Male | 8 | 10 |  |
| Female | 1 | 7 |  |
| Smoking history |  |  | 1 |
| Yes | 4 | 9 |  |
| No | 5 | 8 |  |
| Tumor size（cm） | 2.50±0.77 | 2.15±0.87 | 0.326 |
| Tumor stage |  |  | 1 |
| IA（≤3cm） | 7 | 13 |  |
| IB（＞3cm） | 2 | 4 |  |
| Location of the tumor |  |  | 1 |
| Left lung | 4 | 9 |  |
| Right lung | 5 | 8 |  |
| Tumor pathology |  |  | 1 |
| Adenocarcinoma | 7 | 13 |  |
| Squamous cell carcinoma | 1 | 3 |  |
| Others | 1 | 1 |  |
| Histological grading |  |  | 0.796 |
| G1 | 0 | 1 |  |
| G2 | 4 | 9 |  |
| G3 | 5 | 7 |  |
| TMB（Muts/Mb） | 9.81±9.56 | 7.15±8.19 | 0.463 |
| Driver mutants |  |  |  |
| EGFR | 6 | 10 | 1 |
| TP53 | 6 | 10 | 1 |
| LRP1B | 4 | 4 | 0.514 |
| KRAS | 1 | 4 | 0.809 |
| Ablation power（W） | 31.67±4.33 | 32.35±6.40 | 0.776 |
| Ablation time（min） | 7.00±3.67 | 7.76±2.63 | 0.545 |

**Supplementary Figure 4** Venn diagram integrating ctDNA detection results. The overlap indicates the number of samples with detected ctDNA at both baseline and landmark (= high-risk group), while the number of samples with no ctDNA detected at both baseline and landmark is highlighted above (= low-risk group), number of samples exclusively detected ctDNA either at baseline or landmark (both combined in the medium risk group).


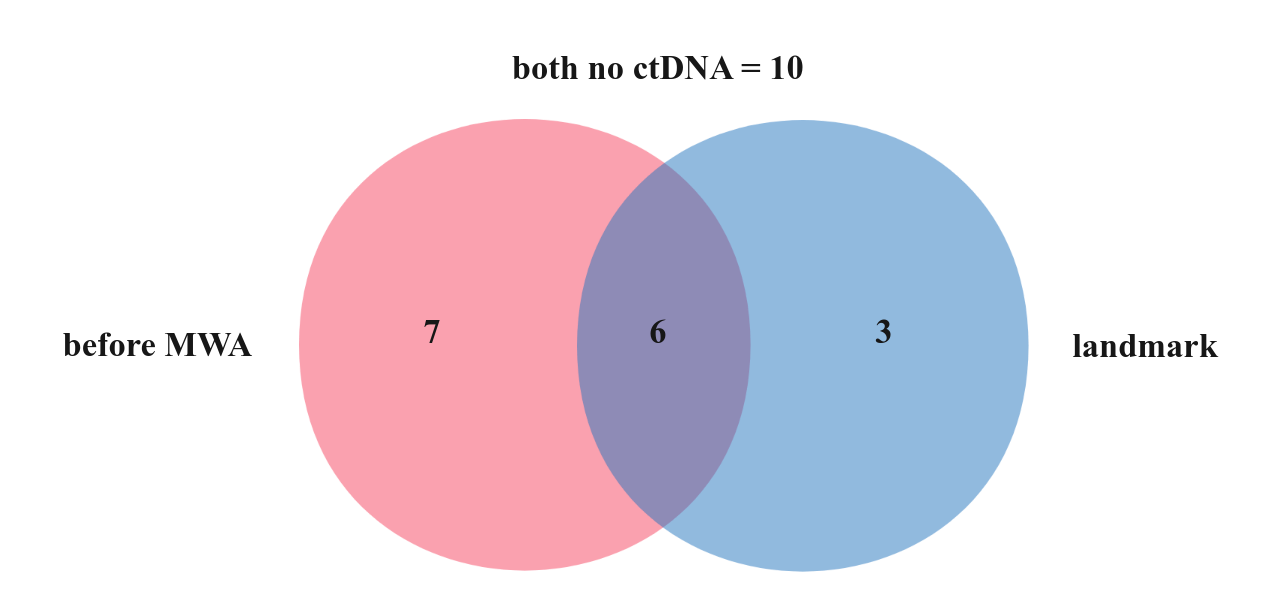


**Supplementary Table 4** The Kaplan-Meier survival analysis of low-risk, medium-risk, and high-risk group.

|  | Group | χ2 | *P* value |
| --- | --- | --- | --- |
| PFS | Low risk | reference |  |
|  | Medium risk | 0.907 | 0.341 |
|  | High risk | 13.280 | 0.000 |

**Supplementary Figure 5** The progression-free survival (PFS) rates of low-risk, medium-risk, and high-risk group.


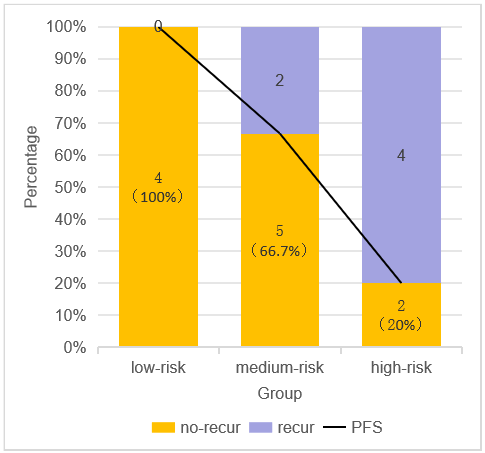


**Supplementary Figure 6** The progression-free survival (PFS) rates of never detected ctDNA, detected ctDNA at least once, and ctDNA persistent groups.


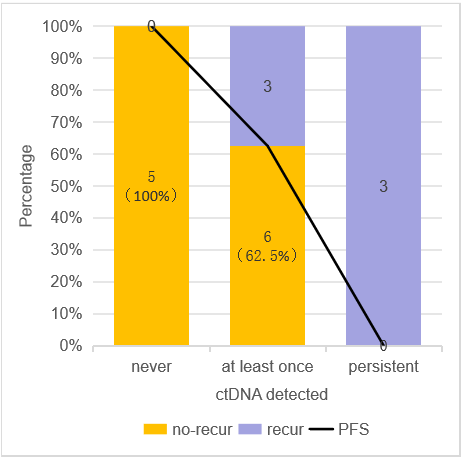


**Supplementary Table 5** The Kaplan-Meier survival analysis of never detected ctDNA, detected ctDNA at least once, and ctDNA persistent groups.

|  | Group | χ2 | *P* value |
| --- | --- | --- | --- |
| PFS | Never detected | reference |  |
|  | Detected at least once | 1.123 | 0.289 |
|  | Persistent | 8.176 | 0.004 |
